# Optimal Recycling Ratio of Biodried Product at 12% Enhances Digestate Valorization: Synergistic Acceleration of Drying Kinetics, Nutrient Enrichment, and Energy Recovery

**DOI:** 10.3390/bioengineering13010109

**Published:** 2026-01-16

**Authors:** Xiandong Hou, Hangxi Liao, Bingyan Wu, Nan An, Yuanyuan Zhang, Yangyang Li

**Affiliations:** 1 Xi’an University of Technology, Xi’an 710049, China; ophion@163.com; 2 Shaanxi Agricultural Development Group, Xi’an 725000, China; 3 State Key Laboratory of Nutrient Use and Management, Key Laboratory of Low-Carbon Green Agriculture, Ministry of Agriculture and Rural Affairs, College of Resources and Environmental Sciences, National Academy of Agriculture Green Development, China Agricultural University, Beijing 100193, China; liaohangxi@163.com (H.L.); sy20243030673@cau.edu.cn (B.W.); sy20243030574@cau.edu.cn (N.A.); yyuanz_00@163.com (Y.Z.)

**Keywords:** recycling ratio of biodried product, drying kinetics, nutrient enrichment, energy recovery, economic-environmental trade-off

## Abstract

Rapid urbanization in China has driven annual food waste production to 130 million tons, posing severe environmental challenges for anaerobic digestate management. To resolve trade-offs among drying efficiency, resource recovery (fertilizer/fuel), and carbon neutrality by optimizing the biodried product (BDP) recycling ratio (0–15%), six BDP treatments were tested in 60 L bioreactors. Metrics included drying kinetics, product properties, and environmental–economic trade-offs. The results showed that 12% BDP achieved a peak temperature integral (514.13 °C·d), an optimal biodrying index (3.67), and shortened the cycle to 12 days. Furthermore, 12% BDP yielded total nutrients (N + P_2_O_5_ + K_2_O) of 4.19%, meeting the NY 525-2021 standard in China, while ≤3% BDP maximized fuel suitability with LHV > 5000 kJ·kg^−1^, compliant with CEN/TC 343 RDF standards. BDP recycling reduced global warming potential by 27.3% and eliminated leachate generation, mitigating groundwater contamination risks. The RDF pathway (12% BDP) achieved the highest NPV (USD 716,725), whereas organic fertilizer required farmland subsidies (28.57/ton) to offset its low market value. A 12% BDP recycling ratio optimally balances technical feasibility, environmental safety, and economic returns, offering a closed-loop solution for global food waste valorization.

## 1. Introduction

China’s urbanization rate has steadily increased to 67% in 2024, up 9.7% from 57.3% in 2015 [1], driving urban population growth from 790 million to 940 million [2]. This has boosted municipal food waste production, which rose from 60 million tons in 2015 to 130 million tons in 2023, with a compound annual growth rate of 7.21% [3]. Consequently, food waste now comprises 30–50% of municipal solid waste, reaching up to 70% in developed regions [4]. Anaerobic digestion, the main food waste treatment, effectively recovers biogas but struggles with large quantities of residual digestate [5]. Digestate accounts for 40–60% of feedstock input in dry anaerobic processes. Based on this range, China’s anaerobic digestion of food waste produced 52–78 million tons of digestate in 2023 [6]. This material exhibits high nutrient and moisture contents. Traditional disposal methods like land application are limited by scarce land, environmental risks, and high transportation costs. Moreover, composted digestate often contains up to 2% salts and heavy metals like copper (Cu) and zinc (Zn), exceeding regulatory limits, necessitating efficient reduction and resource recovery solutions [7].

Biodrying technology uses efficient aeration and microbial heat generation to rapidly reduce the digestate’s moisture content and volume, achieving substantial mass and volume reductions. The resulting biodried product (BDP) has a relatively high calorific value [8], reaching around 1500 kcal/kg. This enables its use as refuse-derived fuel (RDF) for energy recovery via incineration, providing a critical pathway for residues unsuitable for land application. Recycling a portion of BDP as a bulking agent or inoculant for new biodrying feedstock optimizes the process by improving material structure, recycling active microbial communities (accelerating process initiation), enhancing heating and drying rates, and shortening cycles [9]. Consequently, the BDP recycling ratio is a key research parameter, directly influencing drying efficiency, energy consumption, final product properties (nutrient content, calorific value), and operational costs.

Previous studies have explored the technical effects of this ratio. Yu et al. [10] found that recycling 10–15% BDP as a bioactivator enhanced microbial activity and reduced the thermophilic phase by 30%. Yang et al. [11] reported a 22% increase in moisture removal at a 12% ratio, attributed to improved porosity and microbial succession. Chaher et al. [12] noted a trade-off, in which ratios exceeding 20% reduced reactor capacity by 18–25% and decreased total energy recovery. Trzcinski and Stuckey [13] observed that 15% recycling accelerated drying kinetics but reduced the lower calorific value (LHV) by 12% due to moisture retention. Liu et al. [14] used life cycle assessment to emphasize the need for balanced ratios (optimally 8–12%) to reconcile process efficiency and product quality. Most existing studies have focused on the impact of recycling ratios in biological drying on product calorific value and moisture removal. However, there is a lack of systematic comparison of the specific effects of different recycling ratios on the properties of final products (e.g., nutrient retention rate, calorific value improvement efficiency of refuse-derived fuel/compost) as well as the overall environmental–economic performance.

Therefore, this study aims to address these critical gaps. First, biodrying experiments using anaerobic digestate with varying BDP recycling ratios will systematically assess impacts on drying efficiency, key product properties (moisture content, calorific value, nutrient content), and energy consumption to identify optimized parameters. Subsequently, based on these findings, the study will employ detailed Life Cycle Assessment (LCA) and Techno-Economic Analysis (TEA) to comprehensively compare digestate composting and biodrying, quantitatively evaluating their environmental impacts and economic feasibility. The ultimate objective is to provide scientifically robust, data-supported recommendations for environmentally sustainable and economically viable solutions and implementation strategies for treating anaerobic digestion residues from municipal food waste.

## 2. Materials and Methods

### 2.1. Feedstocks and Reactor

Digestate was collected from a high-solids anaerobic digestion (HSAD) reactor, in which the volatile solids (VS) ratio of food waste to inoculum was set at 4:1, and the total solids (TS) content of the mixture was maintained at 20% (*w*/*w*) (Figure S1 shows the schematic diagram of the reactor). The feedstock for biodrying consisted of residual digestate derived from 24-day high-solids mesophilic (35 °C) anaerobic digestion of food waste. Corn stover (CS) was air-dried and mechanically shredded to a particle size of 30–40 mm. The biodried product (BDP) used for recycling was sourced from previous co-biodrying experiments conducted by our group, and it was composed of food waste (FW) anaerobic digestate (66.6% *w*/*w*, wet basis), corn stover (28.6% *w*/*w*, wet basis), and biochar (4.8% *w*/*w*, wet basis). The primary rationale for its incorporation lies in the fixed carbon it contains, which serves as a foundational contributor to the calorific value of the final BDP. Thus, its addition—even when recycled in the form of BDP—directly enhances the total carbon content and higher heating value (HHV) of the initial mixture.

Key physicochemical properties of the initial digestate, corn stover, and recycled BDP are summarized in Table 1.

Biodrying experiments were conducted in custom-built 60 L insulated bioreactors. The reactor vessel consisted of an inner chamber surrounded by an outer jacket filled with insulating material to minimize heat loss. Temperature sensors were inserted into the biomass bed for real-time temperature data logging. An inlet port at the base connected to an air supply system for controlled ventilation. An outlet port at the base for leachate drainage and collection. A top port connected to a condenser tube for vapor discharge and condensation. An additional top port allowed for direct connection to gas sampling bags for periodic exhaust gas analysis.

### 2.2. Experiment Design

The experiment comprised 6 treatments. Treatment 1 (T1) involved the co-biodrying of anaerobic digestate (70%) and corn stover (30%) on a mass basis. The remaining 5 treatments entailed recycling BDP to partially replace corn stover at mass ratios of 10%, 20%, 30%, 40%, and 50%, corresponding to BDP additions of 3%, 6%, 9%, 12%, and 15% relative to the total wet weight of the mixture, respectively. Continuous variable-frequency ventilation was employed for all treatments. The ventilation rate was set to 0.18 L·kg^−1^ DM·min^−1^ for the first 9 days to extend the high-temperature phase, maximize evaporative moisture removal, and reduce energy consumption. For the subsequent 6 days, the ventilation rate was increased to 0.42 L·kg^−1^ DM·min^−1^. This adjustment was justified by the reduced evaporation capacity following the high-temperature phase, with air convection utilized to expedite moisture removal from the system.

### 2.3. Analytical Methods

Temperatures throughout the biodrying process were automatically recorded using temperature sensors installed in the biodrying equipment. Condensed water was quantified daily with an electronic balance. Moisture Content (MC) was determined via the oven-drying method. Daily measurements of O_2_ and CO_2_ concentrations were conducted using a portable gas analyzer (Biogas 5000, Geotech, Leamington Spa, UK). Concentrations of greenhouse gases (CH_4_ and N_2_O) emitted daily during drying were quantitatively analyzed using a gas chromatograph (GC-A90, Echrom, Changsha, China).

Material turning was performed every 3 days. During each turning event, approximately 400 g of representative samples were collected and divided into two portions for subsequent analysis. One portion was preserved fresh to determine moisture content and maturity indices. The other portion was air-dried naturally, ground into a fine powder, and sieved through a 0.15 mm sieve for the analysis of other physicochemical indicators.

In a 150 mL Erlenmeyer flask, 10 g of fresh sample was mixed with 100 mL of deionized water, sealed with gauze, and oscillated in a shaker (GS-10, NANBEI, Zhengzhou, China) for 30 min. After standing for 10 min, the aqueous extract was filtered into a 50 mL Erlenmeyer flask. A 10 cm diameter Petri dish was prepared with filter paper of a matching size and 5 mL of aqueous extract. Ten seeds were evenly placed in the dish and incubated in a culture chamber at 25 ± 1 °C for 48 h. Root lengths were measured, and GI was calculated.

Dried samples were pulverized using a ball mill, sieved through a 100-mesh sieve, and analyzed with an elemental analyzer (vario MACRO cube, Elementar Analysensysteme, Langenselbold, Germany) to test Total Carbon (TC) and Total Nitrogen (TN). Cellulose, hemicellulose, and lignin were determined by the Van Soest method [15]. Starch was measured using the anthrone colorimetric method [16]. Fat was extracted via Soxhlet extraction with diethyl ether [17]. Protein was determined by the Kjeldahl method [18]. Calorific Value was determined via the integral method using a thermogravimetric analyzer (STA7200, Hitachi High-Tech Science, Tokyo, Japan). Nutrient Element Contents in air-dried samples were ball-milled, sieved through a 200-mesh sieve, and analyzed using inductively coupled plasma mass spectrometry (ICP-MS).

All physicochemical property measurements were conducted based on three independent biodrying replicates (*n* = 3). Data points presented in the figures and tables represent the mean values of the three replicates, with error bars indicating the standard deviation (±1 SD). To assess the significance of differences in measured parameters among different treatment groups (T1–T6), One-way Analysis of Variance (One-way ANOVA) was performed for overall comparison. If ANOVA indicated significant differences (*p* < 0.05), Tukey’s Honest Significant Difference (HSD) test was further conducted for pairwise comparisons. All statistical analyses were carried out using SPSS 26.0 software.

### 2.4. Evaluation Indicators

The effective accumulated temperature, biodrying index, moisture removal rate, and water balance were calculated according to Equations (1)–(9).

The effective accumulated temperature reflects the heat accumulation during the process. It is calculated as follows:

(1) $$T_{\mathrm{eff}}=\sum \left(T_{i}-T_{0}\right)\times \Delta t$$

where $T_{i}$ is the pile temperature at time i (°C), $T_{0}$ is the baseline temperature (biological zero point) typically set at 15 °C, and Δt is the duration of the time interval (d).

The biodrying index represents the amount of water removed per unit of organic matter degradation. It is calculated using the following equation:

(2) $$I=\frac{m_{\mathrm{water},\mathrm{removed}}}{m_{\mathrm{OM},\mathrm{consumed}}}$$

where $m_{\mathrm{water}}$ is the total mass of water removed during the process (kg), and $m_{\mathrm{OM}}$ is the total mass of organic matter consumed (kg).

The moisture removal rate indicates the daily efficiency of the condensing system in removing water from the pile:

(3) $$R_{\mathrm{water}}=\frac{m_{\mathrm{condensate}}}{m_{\mathrm{initial}}}$$

where $m_{\mathrm{condensate}}$ is the daily mass of condensate water collected (g d^−1^), and $m_{\mathrm{initial}}$ is the initial mass of the pile (kg).

The water balance describes the dynamics of moisture input and output. Based on the mass conservation law, the moisture loss due to pile turning is calculated as follows:

(4) $$m_{\mathrm{turn}}=\left(m_{\mathrm{initial}}-m_{\mathrm{final}}\right)-m_{\mathrm{evap}}+m_{\mathrm{met}}$$

where $m_{\mathrm{turn}}$ is the moisture loss caused by mechanical turning (kg), $m_{\mathrm{initial}}$ and $m_{\mathrm{final}}$ are the total water mass of the pile at the beginning and end of the process (kg), $m_{\mathrm{evap}}$ is the total evaporated water (kg), and $m_{\mathrm{met}}$ is the water produced by microbial metabolism (kg).

(5) $$m_{\mathrm{water}}=M_{\mathrm{pile}}\times \mathrm{MC}$$

where $m_{\mathrm{water}}$ is the calculated mass of water (kg), $M_{\mathrm{pile}}$ is the total weight of the pile (kg), and MC is the moisture content of the pile (%).

(6) $$m_{\mathrm{evap}}=\int_{t_{i}}^{t_{f}}f\left(t\right)dt$$

where $f\left(t\right)$ is the instantaneous evaporation rate function, and $t_{i}$ and $t_{f}$ represent the start and end times of the ventilation period.

(7) $$f\left(t\right)=Q\times \rho_{\mathrm{air}}\times \frac{H\times M_{w}\times 10^{8.896-\frac{2238}{T+233.8}}}{M_{a}\times P_{\mathrm{atm}}-10^{8.896-\frac{2238}{T+233.8}}}$$

where Q is the airflow rate (m^3^ s^−1^), $\rho_{\mathrm{air}}$ is the air density (kg m^−3^), H is the relative humidity of the exhaust air (%), $M_{w}$ and $M_{a}$ are the molecular weights of water (18) and air (29), $P_{\mathrm{atm}}$ is the standard atmospheric pressure (760 mmHg), and T is the exhaust air temperature (°C).

(8) $$m_{\mathrm{met}}=\Delta \mathrm{BVS}\times C$$

where ΔBVS is the total loss of biodegradable volatile solids (kg), and C is the water production coefficient.

(9) $$m_{\mathrm{evap}}=\left(m_{\mathrm{initial}}-m_{\mathrm{final}}\right)+m_{\mathrm{met}}$$

where $m_{\mathrm{evap}}$ is the total moisture dissipated from the pile (kg), $m_{\mathrm{initial}}$ and $m_{\mathrm{final}}$ are the water masses of the pile at the start and end of the process (kg), and $m_{\mathrm{met}}$ is the water produced by microbial metabolism (kg).

### 2.5. Environmental and Economic Analysis

**Figure 1:**
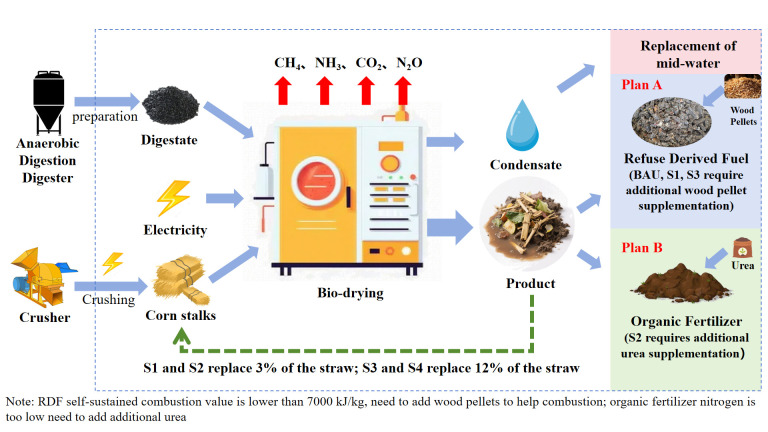
The system boundary of this study.

Environmental impact assessment employs LCA methodology for environmental impact analysis, strictly adhering to the ISO14040 [19] framework [20]. The system boundary encompasses the biodrying process, preparation and utilization of RDF to replace coal combustion, and preparation/application of organic fertilizer substituting chemical fertilizers (Figure 1). The functional unit was defined as treating 10 metric tons of anaerobic digestate/day (wet basis). Key environmental impact categories include global warming potential (GWP100, kg CO_2_-eq), eutrophication potential (EP, t PO_4_^3−^-eq), human toxicity (HT, kg CTUh-eq), and resource depletion (RD, t Sb-eq). Inventory data integrates experimental measurements from this study (capturing nutrient dynamics and heavy metal content under varying BDP recycling ratios) with calculations derived from the GaBi software V10 model.

The economic evaluation classifies costs into investment costs, operational costs, recycling benefits, and product revenue. The primary economic indicator employed is the 20-year Net Present Value (NPV), which enables a comparative analysis of the biodrying and composting pathways across different BDP recycling ratios. Equipment purchase costs and corresponding specifications (i.e., size, capacity, and rated power) were sourced from multiple vendors, previous studies, engineering design models, and the built-in cost model in SuperPro Designer. Material and energy requirements were determined based on mass and energy balance calculations. The total capital investment was a one-time investment in purchasing instruments and equipment.

The annual operation and maintenance cost (OMC) included income tax rate (12.5%), insurance (2.17% PC), equipment maintenance (2.17% PC), labor costs (OL), materials, laboratory (5% of OL), utilities (the annual energy consumption), waste disposal, and depreciation expense in this study. The Labor cost and electricity were set as 4286 USD/year and 0.11 USD/kWh, respectively. The price of materials was assumed to be USD 7.36 and USD 21.43 per dry MT of digestate and corn stover.

Policy scenarios incorporating farmland improvement subsidies (USD 42.86/ton) were assumed in this study. The revenues came from fertilizer/RDF, and condensed water replaces reclaimed water. The costs of RDF and organic fertilizer were set at 164.78 USD/ton and 71.43 USD/ton, respectively. Strict product standards were applied, including that RDF must comply with the CEN/TC 343 standard [21] (requiring an LHV ≥ 7000 kJ/kg). Non-compliant RDF requires blending with auxiliary fuels (e.g., woodchips) to achieve certification. Organic fertilizer must meet the Chinese national standard NY525-2021 (requiring total nutrient content, N + P_2_O_5_ + K_2_O, ≥4%). Non-compliant fertilizer requires supplementation (e.g., with urea) (Table 2).

Five distinct scenarios were established: (1) Baseline Scenario (BAU): Digestate composting without BDP recycling. (2) Scenario A: Optimized Biodrying (RDF): Utilizes the optimal BDP recycling ratio; the resultant product is used as RDF, replacing coal, offering high economic returns but no linkage to agricultural systems. (3) Scenario B: Optimized Biodrying (Safe Agricultural Fertilizer): Utilizes the optimal BDP recycling ratio; the product is processed to meet heavy metal standards and used as safe organic fertilizer, constituting a core pathway for farmland enhancement. This framework facilitates a systematic comparative assessment of technological pathways and product end-uses. It should be noticed that it is assumed in this study that all coefficients are scaled up proportionally based on the 60 L experiment.

## 3. Results

### 3.1. Temperature

**Figure 2:**
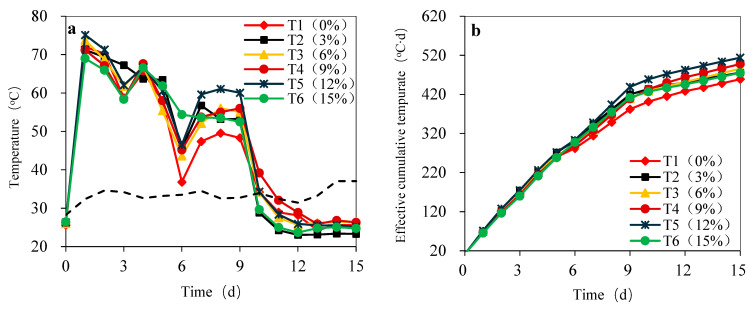
Daily temperature (**a**) and accumulated (**b**) temperature during biodrying of food waste digestate under different biodried product (BDP) recovery ratios. (Note: T denotes the abbreviation of Treatment Group; T1 to T6 represent six experimental groups with BDP recovery ratios of 0%, 3%, 6%, 9%, 12%, and 15%, respectively).

The temperature evolution of the piles displayed a characteristic four-stage pattern: a rapid heating phase, a sustained high-temperature phase, a rapid cooling phase, and a stabilization phase [33] (Figure 2a). During the initial experimental phase (Day 1), all treatments except the high-BDP group (T6, 15%) rapidly reached ultra-high temperatures (>71.19 °C). T5 (12% biodried product) achieved the highest peak temperature of 75.13 °C. This rapid heating is primarily attributed to the swift decomposition of readily degradable organic matter (RDOM) by mesophilic/thermophilic microorganisms, generating significant heat [34]. Conversely, the inadequate temperature increase in T6 was attributed to the dilution of RDOM in the initial mixture, caused by the high BDP recycling ratio. This reduced RDOM proportion limited intense microbial metabolic activity during the early stage.

Following the peak, temperatures fluctuated and gradually declined. Turning operations caused brief temperature rebounds [35]. Notably, although T6 exhibited slower heating, its high-temperature phase duration was relatively longer. This extended duration resulted from the slower consumption rate of the available organic matter [36]. After the first turning, all BDP-amended treatments (T2–T6), except the control (T1), re-entered the high-temperature phase (>50 °C), with T5 demonstrating the best performance. This highlights the key role of thermophilic microbial communities introduced via the BDP in sustaining high-temperature microbial activity. Importantly, all treatments maintained temperatures > 55 °C for over 5 days, satisfying composting hygienic standards [37]. From approximately Day 10 onwards, temperatures dropped rapidly towards ambient levels, indicating the near-exhaustion of RDOM and the onset of the maturation stage.

Accumulated temperature serves as a comprehensive indicator of total biological activity and overall organic matter decomposition during composting [38]. The final effective accumulated temperature differed significantly among treatments: T1 (458.88) < T2 (475.81) < T3 (485.81) < T4 (497.81) < T5 (514.13) > T6 (475.13) (Figure 2b). BDP addition (T2–T5) significantly increased the accumulated temperature compared to the control (T1), confirming its effectiveness in promoting organic matter decomposition and heat generation. This enhancement was accomplished by introducing active microbial communities (recycling effect) and supplementing organic matter, consistent with findings by Díaz-Rodríguez et al. [39]. Accumulated temperature initially increased and then decreased with increasing BDP addition ratio, peaking at T5 (12%). For T1 to T5, the increasing recycling ratio amplified the microbial recycling effect and provided supplementary organic matter (albeit with lower biodegradability), thereby boosting biological activity and cumulative heat production. However, when the ratio reached 15% (T6), the excessive recycling proportion resulted in the dominance of the substrate dilution effect. This significantly reduced the proportion of highly active, fresh RDOM in the initial mixture. Despite the introduced microorganisms, the deficiency of this core “metabolic fuel” limited the intensity and duration of microbial metabolism, failing to support optimal activity. Consequently, the accumulated temperature decreased.

The addition of BDP significantly influenced composting temperature dynamics and organic matter decomposition efficiency via microbial recycling and organic matter supplementation. The optimal addition (12%, T5) achieved the best balance between these two effects. It provided abundant thermophilic microbial inoculum to accelerate the process start-up and sustain high-temperature decomposition activity, while also supplementing available organic matter. Consequently, T5 demonstrated the fastest heating rate, highest peak temperature, most stable high-temperature phase maintenance, and maximum accumulated temperature, representing peak composting efficiency. The performance of T6 (15%) uniquely underscores the critical controlling role of initial RDOM content during the start-up phase. Its slow temperature rise, coupled with a relatively longer high-temperature phase, illustrates how organic matter availability regulates metabolic rate. All treatments met the hygienic requirements for the high-temperature phase. The parabolic relationship (initial increase followed by decrease) between accumulated temperature and addition ratio clearly reveals the key equilibrium point at approximately 12%.

### 3.2. Temperature Germination Index (GI) Dynamics

**Figure 3:**
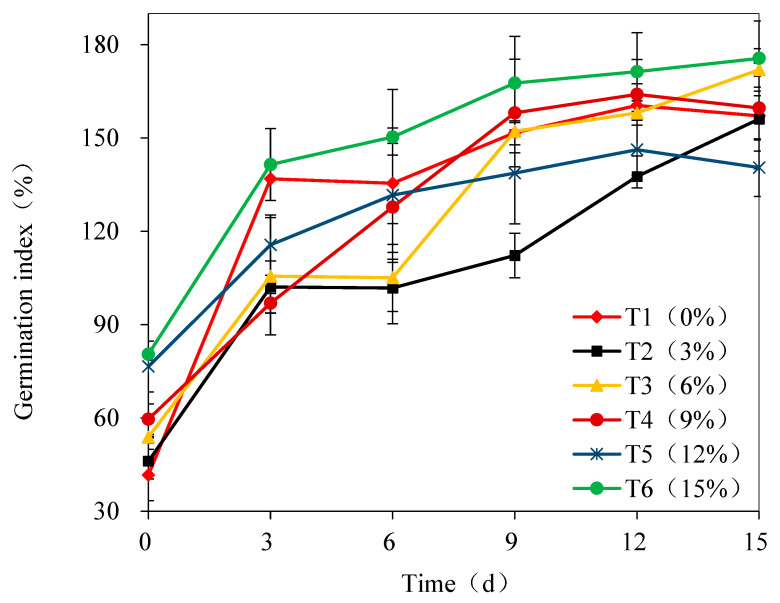
Temporal evolution of germination index (GI) under different recycling rates of biodrying product. Note: Values are presented as mean ± standard deviation (*n* = 3).

The germination index (GI) of all treatments exhibited a gradual upward trend throughout the biodrying process (Figure 3), consistent with the typical patterns of organic matter stabilization and humification during aerobic biodrying [40]. In the initial biodrying stage, only T1 (no BDP addition) and T2 (3% addition) had a GI below 50%. The GI for T3 through T6 was notably higher (>50%) and showed a positive correlation with the recycling ratio of BDP. This effect primarily reflects the maturation recycling effect of the BDP. As highly stabilized and humified materials, the recycled BDP provide an abundance of stable organic matter and functional microbial communities. Increasing their proportion directly enhances the maturity baseline of the initial mixture [41].

By the end of biodrying, the GI for all treatments exceeded 140.5%, far surpassing the widely accepted maturation threshold of 70% [42]. Notably, T6 (15% addition) exhibited the peak GI of 175.6%, 25.0% higher than the lowest value (T5) and averaging 11.8% better than the other treatments (excluding T5). The consistently superior performance of T6 is attributed to its high recycling ratio (15%), which significantly improved the stability of the initial feedstock and reduced phytotoxic substance content, providing an inherent maturity advantage [43]. The mature microbial consortia introduced with the BDP accelerated lignocellulose degradation, promoted humus formation, and likely stimulated the synthesis of plant growth-promoting hormones (e.g., auxins, gibberellins) [44], representing a powerful recycling-driven enhancement of humification.

While some studies have reported that high BDP ratios may reduce the GI due to the accumulation of recalcitrant or inhibitory compounds [45], the peak GI of 175.6% observed in T6 (15% BDP) in this study stems from the unique synergy of anaerobic digestion pretreatment and microbial inoculation–recycling. Unlike prior work, where BDP was derived from untreated or minimally processed feedstocks, our BDP originated from anaerobically digested food waste. AD pretreatment effectively degrades phytotoxic small molecules (e.g., volatile fatty acids, phenols) and partially breaks down complex organics, reducing the inhibitory load of recycled material [46]. This contrasts sharply with Turner et al. [45], who found that a 15% BDP ratio reduced GI by reintroducing untreated inhibitory intermediates; in our case, the pretreated BDP acted as a “maturity amplifier” rather than a contaminant.

### 3.3. Fertilization Indicators

By the end of biodrying (Day 15), total nutrient content (N + P_2_O_5_ + K_2_O) ranged from 3.55% to 4.18%. T6 (15% recycling) attained the highest value (4.18%), representing a 17.7% increase compared to T1 (Table 3). All treatments exhibited significant nutrient enrichment from initial levels (15.4–31.9% increase), confirming that BDP recycling enhances nutrient conservation. These findings align with Raza et al. [47], who attribute such gains to accelerated decomposition via pre-stabilized organic matter and elevated microbial biomass in the inoculum, as well as enhanced microbial colonization and organic matter mineralization facilitated by the inoculum’s high surface area. Notably, at Day 12, T5 (12%) and T6 (15%) reached peak nutrient concentrations (4.19% and 4.20%, respectively), with T5 exceeding China’s organic fertilizer standard (NY 525-2021, ≥4%) [48].

However, extending the process to Day 15 caused nutrient declines in high-inoculum treatments. This phenomenon mirrors Sátiro et al. ’s [49] observations in inoculated composting systems, where prolonged processing under intense microbial activity promoted nutrient losses through ammonia volatilization (nitrogen) and leaching of soluble K ^+^/phosphates [50]. Mechanistically, the initial biodrying phase (Days 1–12) favors the microbial immobilization of nutrients into biomass, while the later phase (Days 12–15) sees a shift toward mineralization and gaseous/liquid losses as readily degradable organic matter becomes scarce [51]. For nitrogen, rising pH (often >8.5 in mature biodrying piles) exacerbates NH_3_ volatilization from ammonium-N [52]; for potassium and phosphorus, increased water-soluble fractions in later stages heighten leaching risk, especially under frequent turning [53].

### 3.4. Separate Balance and Biodrying Index

#### 3.4.1. Moisture Balance

The total moisture removal rates across treatments ranged from 46.2% to 61.4% (Table 4). T1 achieved the highest removal rate (61.44%), a result directly attributable to its significantly lower initial moisture content (48.2%, resulting from a higher proportion of corn stover). This confirms the established principle that initial moisture content is the primary determinant of dehydration potential in biodrying, as lower initial moisture reduces the energy required to reach optimal evaporation thresholds (typically 50–60%) [54]. Evaporation constituted the dominant moisture removal pathway, driven by thermal–microbial synergy, accounting for 89–93% of total water loss (Table 4). The evaporation pattern displayed an initial increase followed by a decrease with increasing BDP proportion. For T2–T5, the enhanced effective accumulated temperature stimulated greater microbial metabolic heat production [55]. This elevated the vapor pressure deficit across the pile material, thereby accelerating evaporation [56]. Mechanistically, mesophilic/thermophilic microbes (e.g., Bacillus, Thermus) in BDP oxidized labile organics, releasing heat that raised pile temperatures to 65–75 °C—optimal for breaking water-holding bonds in organic matrices [57].

Conversely, T6 exhibited the lowest accumulated temperature (475.13 °C·d), stemming from the organic matter dilution effect of the high (15%) recycling ratio. Insufficient heat production limited T6’s evaporative potential. Furthermore, despite the high proportion of mature materials improving pile porosity in T6, the elevated initial moisture content created an excessive absolute moisture load, further impeding evaporation. Turning operations resulted in the loss of 1.14–3.95 kg of bound water released by mechanical disruption. Performing turns during the high-temperature phase (>60 °C) enhanced instantaneous water vapor release. Concurrently, water generated from organic matter degradation was minimal (0.92–1.28 kg). These observations collectively confirm that biodrying operates predominantly through dewatering rather than mineralization. Critically, no leachate was produced in any treatment. This highlights the success of pore structure optimization, achieved particularly through the incorporation of a high proportion of mature materials in T6, in ensuring that moisture migration occurred primarily via the gas phase. This strategy effectively prevented a secondary pollution risk.

#### 3.4.2. The Biodrying Index

**Figure 4:**
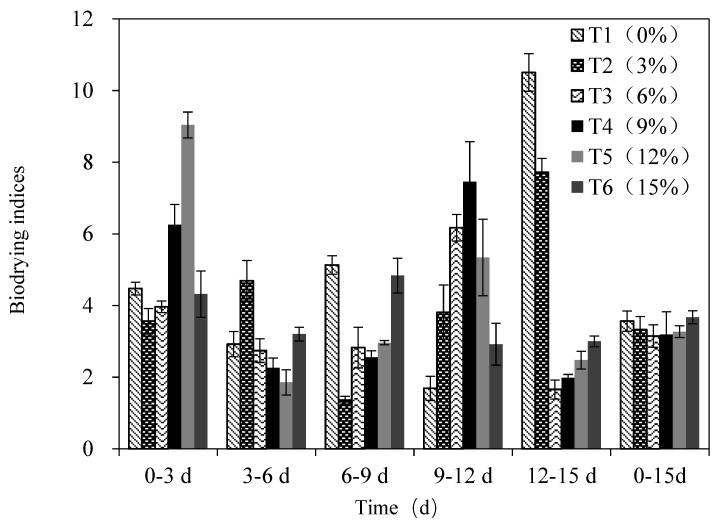
Temporal evolution of biodrying indices under different recycling rates. Note: Values are presented as mean ± standard deviation (*n* = 3).

The biodrying index exhibited distinct, stage-dependent fluctuations. Specifically, values on Day 3 significantly exceeded those observed from Days 4 to 9 (Figure 4). This initial peak is primarily attributed to high-temperature-driven evaporation dominance, during which pile temperatures exceeded 70 °C (Figure 2a). Under these conditions, the moisture evaporation rate, governed by vapor pressure gradients, far surpassed the rate of organic matter degradation. This aligns with the established principle that moisture removal efficiency is maximized during the high-temperature phase. A subsequent rebound in the index after Day 9 correlated directly with enhanced ventilation-assisted mechanical dehydration. Increasing the air volume (typically >0.6 m^3^·kg^−1^·h^−1^) significantly boosted moisture removal capacity through intensified convective airflow, a mechanism critically important during the late stages of food waste drying.

After 15 days, the biodrying index ranged from 3.15 to 3.67. T6 (15% biodried product addition) achieved the highest index (3.67), demonstrating significant optimization. Mechanisms underpinning T6’s success included the high proportion of mature BDP, which optimized pore distribution and gas permeability, significantly improving ventilation-driven dehydration efficiency during the mid-to-late stages. And recycled mature microorganisms maintained high degradation activity, progressively liberating bound water and converting it into free water to augment evaporation potential. This effect was amplified in T6 due to its sustained high-temperature duration. Although high recycling ratios reduce initial organic matter content, their net positive impact results from dual synergistic mechanisms, enhanced anti-compaction properties, air permeability, and prolonged metabolic water release. These combined effects maximize moisture migration efficiency throughout the biodrying process, as empirically demonstrated by T6’s superior performance.

### 3.5. Calorific Value

During biodrying, carbon (C) content decreased significantly in all treatments (Table 5), with initial C contents showing slight fluctuations across different BDP recovery ratios, ranging from 39.23% (T2, 3%) to 41.94% (T3, 6%) and ending at 36.71% (T3, 6%) to 40.00% (T5, 12%), reflecting direct microbial mineralization and decomposition of organic matter. Notably, while initial C content did not exhibit a linear trend with increasing recovery ratios (39.81% in T1 vs. 40.93% in T6), the end C content was generally lower than the initial value except for T2 (39.53% at the end vs. 39.23% initially). This organic matter degradation concomitantly induced a systematic reduction in the Higher Heating Value (HHV), consistent with the established principle linking organic matter loss to energy depletion, initial HHV increased slightly with recovery ratios (14,673.16 kJ·kg^−1^ in T1 to 15,998.39 kJ·kg^−1^ in T6), whereas end HHV peaked at T5 (15,181.07 kJ·kg^−1^, 12%) and declined at T6 (14,751.50 kJ·kg^−1^, 15%). The observed hydrogen (H) reduction is primarily attributable to volatilization of hydrogen-containing gases (e.g., NH_3_, CH_4_) and moisture. The initial H contents remained relatively stable (4.98–5.35%) across all ratios, while end H contents decreased slightly to 4.48–4.95%, with the lowest value in T3 (6%). Conversely, relative enrichment of oxygen (O), nitrogen (N), and sulfur (S) elements stems from aerobic conditions that promote the oxidation of recalcitrant components (e.g., lipid and aromatic polymers) into oxygen-containing functional groups (carboxyl, carbonyl), increasing O content [58]. Specifically, initial O contents decreased marginally from 53.75% (T1) to 52.15% (T6), but end O contents were universally elevated (53.46–56.87%) with the highest enrichment in T3. For N and S, both initial and end contents showed a clear linear increase with increasing BDP recovery ratios: initial N rose from 1.22% (T1) to 1.59% (T6), and end N further increased to 1.45% (T1)–1.83% (T6); initial S increased from 0.24% (T1) to 0.29% (T6), with end S reaching 0.25% (T1)–0.34% (T6). This enrichment is because mineralization rates for organic nitrogen (e.g., proteins) and sulfur-containing amino acids (e.g., cysteine, methionine) lag behind the overall organic matter degradation rate [59]. This differential decay results in the relative concentration of N and S, a phenomenon amplified during food waste drying [60]. Increasing the BDP recycling ratio significantly elevated the initial HHV (from 14,673 to 15,998 kJ·kg^−1^), reflecting the high carbon content and low volatile matter characteristic of mature BDP. However, the initial Lower Heating Value (LHV) simultaneously decreased with higher recycling ratios. This core contradiction arises from the moisture leverage effect; BDP possesses a higher inherent moisture content (~25%) compared to corn stover (~15%), thereby raising the system’s overall moisture. Critically, LHV calculation incorporates a deduction for the latent heat of vaporization associated with this moisture. Despite this initial trade-off, the final LHV after biodrying substantially increased across all treatments (57.4% to 78.4%), achieving values of 4574–5306 kJ·kg^−1^. These values far exceed the self-sustaining combustion threshold (>3350 kJ·kg^−1^). A notable negative correlation emerged between the recycling ratio and the final LHV. Treatments with no (T1) or low (T2, 3%) recycling achieved LHV > 5000 kJ·kg^−1^, meeting RDF preparation standards. In contrast, high recycling ratios (e.g., T6, 15%) yielded a lower final LHV (4574 kJ·kg^−1^), constrained by persistent moisture burden. This demonstrates the critical importance of integrated moisture management. While recycling enhances process maturity and boosts initial HHV, failure to concurrently optimize dehydration (e.g., by adding high-water-absorbing conditioners) allows inherent moisture disadvantages to limit the achievable LHV energy potential.

### 3.6. Environmental and Economic Assessment

#### 3.6.1. Environmental Indicator Analysis

**Figure 5:**
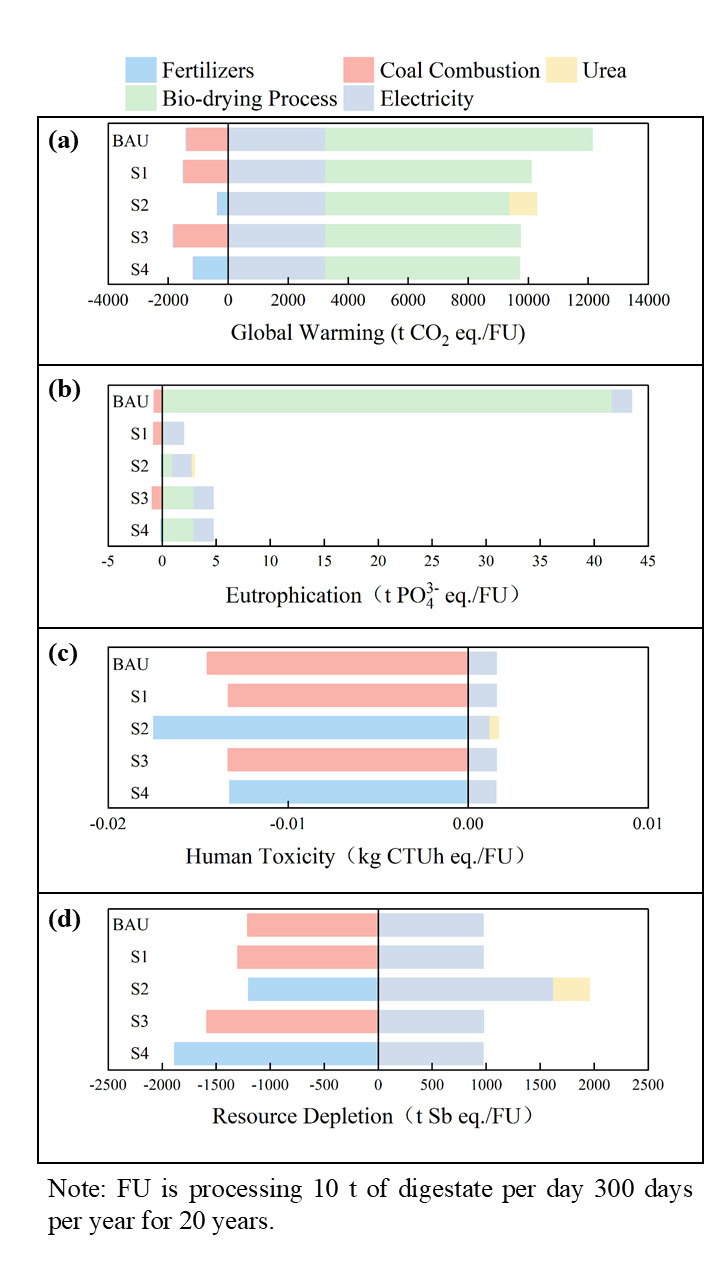
The global warming potential (**a**), eutrophication potential (**b**), human toxicity (**c**), and resource depletion (**d**) of five scenarios.

The life cycle assessment revealed significant trade-offs in global warming potential (GWP), eutrophication potential (EP), human toxicity (HT), and resource depletion (RD, t Sb-eq) across the five scenarios, critically influenced by the degree of digestate recycling (3% vs. 12%) and the final product strategy (RDF vs. Organic Fertilizer—OF) (Figure 5).

The BAU exhibited the highest GWP, primarily driven by emissions from the biodrying process itself. Implementing digestate recycling significantly reduced GWP across all scenarios. The lowest GWP was achieved by S4 (12% recycling, OF) at 6475.9 t CO_2_-eq/FU, representing a 27.3% reduction compared to BAU. This substantial decrease stems from the displacement of coal combustion in external processes, analogous to findings by Wang et al. [61] on process energy substitution benefits, and the avoidance of synthetic fertilizer production, consistent with the well-established high carbon footprint of urea/N-fertilizers. While S3 (3% recycling, OF) also showed significant GWP reduction, S4’s lower value confirms that higher recycling ratios enhance the system’s ability to internally utilize organic matter, minimizing direct emissions and maximizing displacement credits. Interestingly, S2 (12% recycling, RDF) also performed well, demonstrating that high recycling can effectively mitigate GWP even within an RDF pathway, mainly by reducing the biodrying process emissions compared to S1 and BAU.

The most dramatic impact was observed on gas emissions during biodrying. The BAU showed alarmingly high EP (41.7 t PO_4_-eq/FU), likely due to uncontrolled nitrogen volatilization (as NH_3_). Both recycling and the OF product pathway drastically mitigated this. S1 (3% recycling, OF) achieved a remarkable 99.5% reduction, while S4 also maintained very low levels. This aligns strongly with studies emphasizing process control and product stabilization for N management [62]. However, S2 exhibited higher EP than S1 and S4, attributable to the addition of urea, likely needed to meet RDF calorific value specifications, introducing a new eutrophication burden. This highlights a critical trade-off for RDF production, where efforts to boost energy content can inadvertently increase environmental burdens elsewhere, a challenge noted in RDF quality standardization discussions.

Impacts in the HT and RD categories were relatively low across all scenarios. However, S4 consistently showed the lowest or among the lowest HT values, reinforcing the OF pathway’s advantage in minimizing toxic burdens. RD was significantly reduced in S2 compared to BAU, primarily due to the avoided coal consumption. This substitution benefit, replacing fossil fuels with biomass-derived RDF, is a key argument for waste-to-energy pathways, though its climate benefit (GWP reduction) in S2 was less pronounced than in the OF pathways (S3 and S4) due to the higher process emissions inherent in RDF production observed here. The OF scenarios (S3 and S4) also achieved substantial fossil depletion reductions, demonstrating multiple pathways for resource conservation.

Notably, the interaction between BDP recovery ratios (high: 12–15% vs. low: 3%) and product pathways (organic fertilizer vs. RDF) jointly determines the physicochemical properties of the material (e.g., calorific value, nutrient content), thereby inducing differences in environmental indicators. High BDP ratios significantly reduce direct emissions and resource consumption by optimizing microbial activity and pore structure; they also inhibit nitrogen volatilization to lower eutrophication potential (EP), although the addition of urea in the RDF pathway conversely exacerbates EP fluctuations. Furthermore, high BDP ratios promote nutrient enrichment (with N + P_2_O_5_ + K_2_O reaching 4.19%) but may constrain energy recovery due to moisture retention. Among the scenarios, S4 (12% BDP + organic fertilizer pathway) achieves maximized avoided-emission credits through the substitution effect of organic matter internal circulation for fossil fuels and synthetic fertilizers, emerging as the scenario with the lowest global warming potential (GWP: 6475.9 t CO_2_-eq). In contrast, low BDP ratios (3%), while lacking the aforementioned environmental and nutrient advantages, enhance the material’s lower heating value (LHV > 5000 kJ·kg^−1^), making it more compatible with the energy recovery requirements of the RDF pathway. This characteristic also shapes S2 (12% BDP + RDF pathway): despite a higher GWP than S4 due to moisture retention constraints and the addition of urea. In comparison, S4, being highly aligned with the nutrient demands of the organic fertilizer pathway, stands out as the optimal choice for environmental performance.

#### 3.6.2. Analysis of Economic Indicators

The economic analysis, as summarized in Table 6, reveals significant variations in profitability across scenarios. The BAU yields a negative NPV of −USD 35,418.7, indicating economic infeasibility under baseline conditions. Both RDF-oriented scenarios (S1 and S2) show positive and substantially higher NPVs, USD 202,812.9 for S1 and USD 716,725.5 for S2, demonstrating that higher recycling ratios enhance profitability when producing RDF. Scenarios producing RDF (BAU, S1, S2) demonstrated significantly stronger economic performance under current market conditions. S2 (12% recycling, RDF) emerged as the most economically viable option, yielding a compelling NPV of USD 716,725.5. This superiority stems from a higher RDF market value. And S2 generated the highest annual revenue, driven by high RDF sales volume and efficient raw material cost reduction; corn stover costs plummeted to USD 16,531/year due to the 12% digestate recycling substituting virgin feedstock. While wood chip pellet costs increased, likely needed for blend optimization, the net effect on profitability was strongly positive. This demonstrates the economic leverage of high recycling ratios in RDF production, optimizing feedstock use. S1 (3% recycling, RDF) also showed a positive NPV (USD 202,812.9), significantly outperforming BAU, confirming the economic benefit of even modest recycling. Within the RDF pathway, increasing the recycling ratio from 3% (S1) to 12% (S2) dramatically improved NPV by over 250%. These underscores recycling not just as an environmental mitigation strategy, but as a powerful economic lever within waste valorization systems, enhancing resource efficiency and product value.

For organic fertilizer (OF)-oriented scenarios, S3 results in a highly negative NPV of -USD 805,040.3, primarily due to low OF pricing and high operational costs. In contrast, S4 achieves a positive NPV of USD 304,770.4, suggesting that a higher recycling ratio can make OF production economically viable, especially when combined with farmland improvement subsidies. Operational costs are lowest in S3 and S4, primarily due to reduced raw material costs. However, revenue streams differ markedly; RDF scenarios benefit from higher market prices (up to USD 42.6/ton), while OF scenarios rely heavily on subsidies and lower-value product sales. In stark contrast, the economic performance of OF scenarios (S3, S4) was highly sensitive to product value and policy support. S4 (12% recycling, OF) suffered a severely negative NPV, primarily due to the low market price of OF. This price is insufficient to cover costs, despite S4 achieving the lowest environmental footprint (GWP). This disconnect between high environmental benefit and low market value is a well-documented challenge for organic waste-derived fertilizers. S3 (3% recycling, OF), however, achieved a positive NPV. The critical difference is the farmland improvement subsidies, which effectively bridged the economic gap for S3. This highlights the essential role of policy instruments (subsidies, carbon credits, fertilizer taxes) in making environmentally superior OF pathways viable, aligning with calls for internalizing environmental externalities. Without such intervention (as in S4), the OF pathway is economically unsustainable under the modeled conditions, despite its environmental merits. The reclaimed water revenue provided a minor, consistent benefit across all scenarios.

It should be noticed that the LCA and TEA result was based on the 60 L small-scale insulated reactor, which offers controllable heat loss (small surface area-to-volume ratio), feasible uniform bottom aeration, and maintainable material homogeneity via regular turning. However, scale-up introduces three key challenges: (1) increased heat loss (surface area outpacing volume) requires more external energy, raising LCA’s GWP (fossil fuel-dependent) and TEA’s O and M costs, mitigated by high-efficiency insulation and waste heat recovery; (2) poor aeration uniformity (risking anaerobic pockets/uneven drying) drives disproportionate energy use, impacting TEA costs and LCA’s electricity-related burdens, addressed via CFD-optimized air distribution; (3) pronounced temperature/moisture/microbial gradients in large silos cause uneven drying/excessive mineralization, compromising product quality and introducing LCA/TEA uncertainties, resolved by online monitoring and process control.

#### 3.6.3. Nutrient Enrichment vs. Fuel Quality Trade-Off

This study quantifies the significant interplay between process design (recycling ratio), product strategy, and policy in determining the environmental and economic sustainability of biodrying systems. Our findings on the GWP benefits of digestate recycling and coal/fertilizer displacement corroborate life cycle studies on advanced biological treatments [63]. The dramatic reduction in EP via controlled biodrying and OF production aligns with research emphasizing closed-loop nutrient management [64]. However, the identification of the addition of urea as an EP hotspot for high-quality RDF production adds a crucial nuance often overlooked in RDF LCA studies focused primarily on GWP.

Economically, the superior viability of the RDF pathway under current market conditions mirrors trends in regions with established waste-derived fuel markets [65]. The paramount importance of subsidies for OF viability resonates strongly with analyses of biofertilizer markets in developing economies, where low willingness-to-pay hinders adoption [66]. Our results emphasize that high recycling ratios (12%) can be a key differentiator for economic success in RDF production, optimizing costs and potentially enhancing product quality/value, a finding that extends previous work focusing mainly on process stability.

While the RDF pathway (particularly S2) offers the best economic return under prevailing market conditions, the OF pathway (S4) delivers superior environmental performance, especially in GWP reduction. This creates a sustainability dilemma. Policy intervention, such as the subsidies enabling S3’s viability or mechanisms valuing carbon sequestration/fertilizer displacement, is crucial to align economic incentives with environmental benefits and unlock the full potential of OF pathways. Future system optimization should focus on maximizing recycling ratios for resource efficiency, minimizing nitrogen losses during biodrying (especially for RDF), and developing robust policy frameworks that recognize and reward the environmental services provided by high-quality organic fertilizers derived from waste.

## 4. Conclusions

Recycling BDP as a bioactivator significantly improved biodrying performance. The 12% ratio optimized microbial heat generation and organic matter degradation, shortening the drying cycle to 12 days. Moisture removal reached 61.4% in T1 (0% BDP), but 12% BDP achieved the highest biodrying index (3.67) through pore-structure optimization and sustained microbial activity. At 12% BDP, total nutrients (N + P_2_O_5_ + K_2_O) peaked at 4.19% by Day 12, complying with organic fertilizer standards. Conversely, ≤3% BDP maximized LHV, meeting RDF energy thresholds. Higher ratios (15%) reduced the net calorific value by 12% due to inherent moisture in BDP, highlighting that BDP recycling favors fertilizer production over energy recovery. The RDF pathway with 12% BDP recycling yielded the highest NPV (716,725). For organic fertilizer, subsidies were essential to achieve positive NPVs, as low OF prices cannot offset costs despite superior GI and low GWP. Prioritize ≤3% BDP for regions with established waste-to-energy markets. Adopt 12–15% BDP with policy incentives to leverage agronomic benefits.

## Figures and Tables

**Table 1:** Physical and chemical characteristics of feedstocks.

Determination Indicators	Anaerobic Digestate	Corn Stover	BDP
Moisture content (%) ^a^	85.60 ± 0.12	6.56 ± 0.17	34.39 ± 0.01
Volatile matter (%) ^b^	74.61 ± 0.15	94.05 ± 0.28	83.56 ± 0.09
Cellulose (%) ^b^	20.16 ± 0.15	33.39 ± 0.14	27.36 ± 0.17
Hemicellulose (%) ^b^	9.32 ± 0.17	25.51 ± 0.36	14.52 ± 0.20
Lignin (%) ^b^	12.20 ± 0.07	2.94 ± 0.10	7.98 ± 0.21
Starch (%) ^b^	1.94 ± 0.15	1.27 ± 0.19	0.94 ± 0.11
Fat (%) ^b^	6.30 ± 0.11	5.82 ± 0.15	7.04 ± 0.30
Crude protein (%) ^b^	9.62 ± 0.38	4.57 ± 0.12	9.32 ± 0.15
Total carbon (%) ^b^	36.06 ± 0.37	43.66 ± 0.20	43.00 ± 0.20
Total nitrogen (%) ^b^	1.60 ± 0.10	0.78 ± 0.08	1.61 ± 0.09
pH	8.46 ± 0.03	6.11 ± 0.02	7.70 ± 0.04
Higher heating value (kJ·kg^−1^) ^b^	14,418.70 ± 170.30	14,195.71 ± 96.09	16,985.23 ± 156.90
Lower heating value (kJ·kg^−1^) ^b^	−973.93 ± 66.69	11,817.42 ± 39.18	9602.40 ± 30.11

Note: ^a^ is based on wet weight; ^b^ is based on dry weight. Values are presented as mean ± standard deviation (*n* = 3).

**Table 2:** Economic evaluation parameters for 5 scenarios.

Parameters	Unit	Numerical Value	Economic Parameters	Unit	Numerical Value	Reference
***Time parameters***			***Raw materials***			
Analyze the year	-	2024	Digestate	$/t	7.36	[22]
Project lifespan	Year	20	Corn stover	$/t	21.43	[23]
Annual operating time	Hour	7200	Nitrogen price *****	$/t	257.14	[24]
***Financing parameters*** *			Wood piece	$/t	44.33	[25]
Depreciation method	-	Straight-line method	***Utilities consumption***			
Depreciation period	Year	10	Electricity	$/kWh	0.11	[26]
Depreciation rate	%	5	***Revenue***			
Inflation rate	%	8	RDF	$/t	164.78	[27]
Income tax rate	%	12.5	Organic fertilizer	$/t	71.43	[28]
***Total capitalized cost (TCC)*** **	%PC ***	100	Carbon price	$/t CO_2_-eq	15.00	[29]
***Labor***	$/year/person	4286 ****	Farmland improvement subsidies	$/t	42.86	[30]
			Condensate	$/ton	0.26	[31]

Note: * The data was collected from [32]. ** Total capitalized cost included total direct costs (135% PC), total indirect costs (54% PC), contractor fees (9.45% PC), startup costs (9.45% PC), and emergencies (18.9% PC). *** PC: The cost of equipment acquisition (Plant Cost). **** The data was collected from the Chinese Market. ***** The price of this data is calculated based on the price of urea, which is 118 USD/ton.

**Table 3:** Temporal evolution of total nutrient content under different recycling rates of biodrying product.

Treatment	N			P_2_O_5_			K_2_O			Total Nutrients (N + P_2_O_5_ + K_2_O)		
	D0	D12	D15	D0	D12	D15	D0	D12	D15	D0	D12	D15
T1 (0%)	1.22 ± 0.01	1.34 ± 0.00	1.45 ± 0.03	0.54 ± 0.03	0.52 ± 0.02	0.60 ± 0.00	1.31 ± 0.01	1.33 ± 0.01	1.50 ± 0.01	3.07 ± 0.05	3.20 ± 0.04	3.55 ± 0.04
T2 (3%)	1.23 ± 0.00	1.45 ± 0.01	1.59 ± 0.02	0.59 ± 0.04	0.59 ± 0.03	0.57 ± 0.02	1.34 ± 0.05	1.44 ± 0.00	1.40 ± 0.03	3.35 ± 0.08	3.48 ± 0.04	3.57 ± 0.07
T3 (6%)	1.24 ± 0.03	1.52 ± 0.04	1.65 ± 0.00	0.48 ± 0.00	0.57 ± 0.01	0.69 ± 0.03	1.22 ± 0.03	1.44 ± 0.04	1.53 ± 0.02	2.94 ± 0.06	3.54 ± 0.09	3.87 ± 0.05
T4 (9%)	1.36 ± 0.01	1.66 ± 0.01	1.86 ± 0.03	0.54 ± 0.01	0.64 ± 0.01	0.72 ± 0.02	1.23 ± 0.01	1.50 ± 0.01	1.50 ± 0.00	3.13 ± 0.03	3.80 ± 0.03	4.08 ± 0.05
T5 (12%)	1.37 ± 0.00	1.71 ± 0.03	1.78 ± 0.01	0.55 ± 0.02	0.81 ± 0.04	0.69 ± 0.05	1.24 ± 0.08	1.66 ± 0.06	1.50 ± 0.04	3.17 ± 0.11	4.19 ± 0.14	3.97 ± 0.10
T6 (15%)	1.59 ± 0.01	1.90 ± 0.00	1.83 ± 0.02	0.68 ± 0.03	0.71 ± 0.00	0.72 ± 0.00	1.33 ± 0.01	1.59 ± 0.06	1.63 ± 0.09	3.60 ± 0.05	4.20 ± 0.06	4.18 ± 0.09

Note: All the processed and determined indicators are based on the dry basis. Values are presented as mean ± standard deviation (*n* = 3).

**Table 4:** Water mass balance under different recycling rates of biodrying product.

Treatment	T1 (0%)	T2 (3%)	T3 (6%)	T4 (9%)	T5 (12%)	T6 (15%)
Evaporation removing water (kg)	6.53	7.04	7.14	7.16	7.52	6.63
Loss of moisture, such as by turning the pile (kg)	3.95	3.39	3.32	1.19	1.14	2.55
Water production from organic matter degradation (kg)	1.12	1.14	1.28	0.95	0.97	0.92
Total water removal amount (kg)	9.35	9.29	9.18	7.39	7.70	8.26
Total moisture removal (%)	61.44	58.65	56.93	46.18	47.09	49.50

Note: No leachate is generated in all treatments. Values are presented as mean ± standard deviation (*n* = 3).

**Table 5:** The ultimate analysis and heating value of the initial and end product under different recycling ratios.

Determination Indicators	Time	T1 (0%)	T2 (3%)	T3 (6%)	T4 (9%)	T5 (12%)	T6 (15%)
C (%)	Initial	39.81	39.23	41.94	41.55	40.63	40.93
	End	38.71	39.53	36.71	38.66	40.00	39.61
H (%)	Initial	4.99	4.98	5.35	5.24	5.04	5.05
	End	4.92	4.95	4.48	4.65	4.77	4.76
O (%)	Initial	53.75	54.30	51.25	51.58	52.70	52.15
	End	54.68	53.65	56.87	54.50	53.13	53.46
N (%)	Initial	1.22	1.23	1.24	1.36	1.37	1.59
	End	1.45	1.59	1.65	1.86	1.78	1.83
S (%)	Initial	0.24	0.26	0.22	0.27	0.26	0.29
	End	0.25	0.28	0.30	0.33	0.32	0.34
HHV (kJ·kg^−1^)	Initial	14,673.16	14,835.48	14,922.61	15,370.29	15,790.29	15,998.39
	End	12,950.02	13,727.11	13,701.32	14,361.69	15,181.07	14,751.50
LHV (kJ·kg^−1^)	Initial	3019.67	2939.30	2746.49	2785.79	2825.65	2743.92
	End	5306.12	5004.37	4898.77	4574.35	4485.06	4318.76

Note: All the processed and measured indicators are based on the dry basis; in the table, the standard deviation range of C content is 0.15–1.22%, the standard deviation range of H content is 0.05–0.18%, the standard deviation range of N content is 0.00–0.02%, the standard deviation range of S content is 0.01–0.02%, the standard deviation range of high calorific value is 123.88–377.13 kJ∙kg^−1^, and the standard deviation range of low calorific value is 23.07–118.09 kJ∙kg^−1^.

**Table 6:** Analysis of profitability in different scenarios.

Category	Unit	BAU	S1	S2	S3	S4
***Total capitalized cost***	USD	453,600	453,600	453,600	453,600	453,600
**Raw material cost**	USD	58,790.7	56,711.6	56,585.0	46,875.6	38,610.6
Digestate	USD	22,080.0	22,080.0	22,080.0	22,080.0	22,080.0
Corn stover	USD	27,550.9	24,795.6	16,530.6	24,795.6	16,530.6
wood chips pellets	USD	7169.5	9835.9	16,488.1	-	-
Supplement N	USD	1990.3	-	1486.2	-	-
**Utilities**	USD	31,600.8	31,600.8	31,600.8	31,600.8	31,600.8
**Labor**	USD	8572.0	8572.0	8572.0	8572.0	8572.0
**Laboratory**	USD	1285.8	1285.8	1285.8	1285.8	1285.8
**Insurance**	USD	3.0	3.0	3.0	3.0	3.0
**Equipment maintenance**	USD	3.0	3.0	3.0	3.0	3.0
**Income taxes**	USD	19,772.4	22,997.4	30,553.6	242.1	13,296.0
***Operation***	USD	120,027.7	121,173.6	128,603.2	88,582.4	93,371.2
***Revenue***	USD	158,178.9	183,978.9	244,428.9	127,854.2	169,750.8
RDF	USD	157,318.0	183,154.0	243,699.9		
Organic fertilizer	USD	-	-	-	79,393.3	105,638.6
Farmland improvement subsidies	USD	-	-	-	47,636.0	63,383.2
Alternative reclaimed water	USD	860.8927255	824.9116528	728.9782071	824.9116528	728.9782071
***Economic analysis***						
NPV (interest rate 8.0%)	USD	−35,418.7	202,812.9	716,725.5	−141,061.3	304,770.4
The price of RDF	USD	169.2	143.3	107.6	-	-
The price of OF	USD	-	-	-	86.4	47.1

## Data Availability

Due to the nature of this research, participants of this study did not agree for their data to be shared publicly, so supporting data is not available.

## Supplementary Materials

The following supporting information can be downloaded at: https://www.mdpi.com/article/10.3390/bioengineering13010109/s1, Figure S1. Diagram of biodrying reactor. Table S1. The Ph, EC, E4/E6 under different inoculation radio of biobiodrying product.
