# Supplementary material for: Optimal Recycling Ratio of Biodried Product at 12% Enhances Digestate Valorization: Synergistic Acceleration of Drying Kinetics, Nutrient Enrichment, and Energy Recovery
Source: Bioengineering (Basel). 2026 Jan 16;13(1):109. doi: 10.3390/bioengineering13010109 (PMC12837798; doi:10.3390/bioengineering13010109)
Supplement: Supplementary file 1 [file bioengineering-13-00109-s001.zip › bioengineering-4070585-supplementary.pdf]

Supplementary Material

Optimal Recycling Ratio of Biodried Product at 12% Enhances Digestate Valorization: Synergistic Acceleration of Drying Kinetics, Nutrient Enrichment, and Energy Recovery

Summary

Figure S1 Diagram of biodrying reactor.
Table S1 The Ph、EC、E_4/E_6 under different inoculation radio of biobiodrying product.

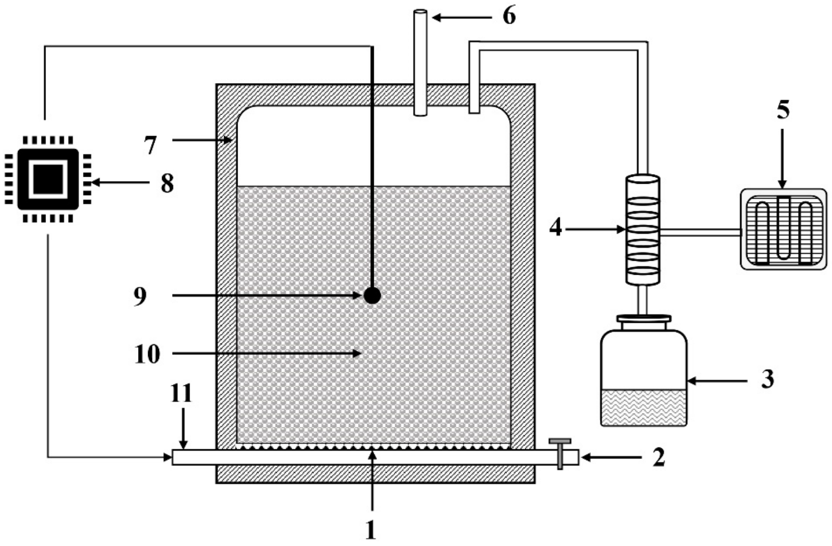

1. Sieve plate; 2. Leachate sampling port; 3. Bottle for evaporated water collection; 4. Condenser pipe; 5. Refrigerator; 6. Gas outlet sampling; 7. Heat insulating layer; 8. Automatic control system; 9. Temperature sensor; 10. Substrates; 11. Ventilated place

Fig. S1. Diagram of biodrying reactor

Table S1

The Ph、EC、E_4/E_6 under different inoculation radio of biobiodrying product.

Treatment	Biodrying period	pH	EC	E_4/E_6
		-	mS·cm^{-1}	-
T1(0%)	Initial	7.62 ± 0.04	1.89 ± 0.02	3.83 ± 0.13
	End	7.32 ± 0.03	1.82 ± 0.01	2.54 ± 0.11
T2(3%)	Initial	7.72 ± 0.06	1.88 ± 0.12	3.76 ± 0.00
	End	7.26 ± 0.04	1.86 ± 0.02	2.67 ± 0.10
T3(6%)	Initial	7.89 ± 0.02	1.74 ± 0.04	3.43 ± 0.00
	End	7.27 ± 0.01	1.83 ± 0.03	2.35 ± 0.00
T4(9%)	Initial	8.03 ± 0.10	1.72 ± 0.07	3.44 ± 0.03
	End	7.29 ± 0.00	1.53 ± 0.02	2.68 ± 0.03
T5(12%)	Initial	8.08 ± 0.04	1.65 ± 0.06	3.21 ± 0.03
	End	7.39 ± 0.04	1.40 ± 0.13	2.68 ± 0.09
T6(15%)	Initial	8.12 ± 0.07	1.53 ± 0.18	3.14 ± 0.00
	End	7.38 ± 0.05	1.51 ± 0.03	1.76 ± 0.14
